# The relationship between triglyceride-glucose index and prospective key clinical outcomes in patients hospitalised for coronary artery disease

**DOI:** 10.1186/s12933-024-02132-2

**Published:** 2024-01-22

**Authors:** Benchuan Hao, Lyu Lyu, Juan Xu, Xiaoqing Zhu, Cui Xu, Weiyang Gao, Ji Qin, Taoke Huang, Yipu Ding, Ziyue Zhang, Yanhui Yang, Hongbin Liu

**Affiliations:** 1 Department of Cardiology, The Second Medical Center, Chinese PLA General Hospital, Beijing, China; 2 Department of General Surgery, Affiliated Xiaoshan Hospital, Hangzhou Normal University, Hangzhou, China; 3 Medical School of Chinese PLA, Beijing, China; 4 Department of Medical Administration, The 305 Hospital of PLA, Beijing, China; 5 Outpatient Department, Hospital of PLA, Hanzhong, China

**Keywords:** Triglyceride-glucose index, Coronary heart disease, All-cause mortality, Major adverse cardiovascular events, Nonlinear association

## Abstract

**Background:**

The triglyceride-glucose (TyG) index is regarded as a dependable alternative for assessing insulin resistance (IR), given its simplicity, cost-effectiveness, and strong correlation with IR. The relationship between the TyG index and adverse outcomes in patients with coronary heart disease (CHD) is not well established. This study examines the association of the TyG index with long-term adverse outcomes in hospitalized CHD patients.

**Methods:**

In this single-center prospective cohort study, 3321 patients hospitalized with CHD were included. Multivariate Cox regression models were employed to assess the associations between the TyG index and the incidence of all-cause mortality and major adverse cardiovascular events (MACEs). To examine potential nonlinear associations, restricted cubic splines and threshold analysis were utilized.

**Results:**

During a follow-up period of 9.4 years, 759 patients (22.9%) succumbed to mortality, while 1291 (38.9%) experienced MACEs. Threshold analysis demonstrated a significant “U”-shaped nonlinear relationship with MACEs, with different hazard ratios observed below and above a TyG index of 8.62 (below: HR 0.71, 95% CI 0.50–0.99; above: HR 1.28, 95% CI 1.10–1.48). Notably, an increased risk of all-cause mortality was observed only when the TyG index exceeded 8.77 (HR 1.53, 95% CI 1.19–1.96).

**Conclusions:**

This study reveals a nonlinear association between the TyG index and both all-cause mortality and MACEs in hospitalized CHD patients with CHD. Assessing the TyG index, particularly focusing on individuals with extremely low or high TyG index values, may enhance risk stratification for adverse outcomes in this patient population.

**Supplementary Information:**

The online version contains supplementary material available at 10.1186/s12933-024-02132-2.

## Introduction

Coronary heart disease (CHD) represents a prevalent form of cardiovascular disease and a leading cause of mortality globally, exerting a considerable burden on public health worldwide [1]. Exploring predictive markers for risk stratification in patients with CHD is crucial to mitigate adverse outcomes.

Insulin resistance (IR), often occurring years before the onset of type 2 diabetes (T2DM), is characterized by a reduced response to insulin, diminishing its effectiveness [2]. IR plays a central role in the early stages of T2DM and is closely associated with the onset and progression of CHD [3]. Insulin resistance, as an abnormal metabolic status, can affect the levels of various metabolic markers in the body, including triglycerides and glucose. Simental et al. [4] first discovered a strong correlation between the triglyceride-glucose (TyG) index, obtained by normalizing the product of fasting glucose and triglycerides, and insulin resistance, surpassing the correlation observed with the homeostasis model assessment-estimated insulin resistance index. The TyG index also exhibits better predictive performance in identifying diabetic patients than when using fasting glucose and triglycerides separately [5]. Subsequent extensive research has confirmed TyG index as a cost-effective and convenient alternative marker of insulin resistance [6].

In the past decade, numerous studies have been conducted to ascertain if the TyG index can act as an alternative indicator of IR in stratifying the risk for adverse outcomes. The TyG index is not only associated with the occurrence of cardiovascular disease (CVD) [7–15] but also with adverse outcomes in patients with CVD [16–23]. Numerous studies have independently confirmed the association of the TyG index with adverse outcomes in CHD patients, both with and without T2DM [18, 24, 25]. However, many studies have yielded conflicting results [26, 27]. Recent research has also suggested a potential “U”-shaped nonlinear association between the TyG index and mortality in patients with CVD [28, 29], as well as the incidence of CVD in older individuals [30].

The inconsistent conclusions from these studies highlight the need for further research to confirm the relationship between the TyG index and adverse cardiovascular events. Currently, there is a lack of comprehensive cohort studies supporting the predictive value of the TyG index for adverse outcomes over a long period in hospitalized patients with CHD, especially concerning the potential nonlinear association between the TyG index and these outcomes. This study aims to examine the association between the TyG index and adverse outcomes over an extended period in hospitalized patients with CHD and to explore potential nonlinear associations.

## Methods

### Study design and participants

This was a prospective cohort study conducted from October 2010 to September 2014. A total of 3,670 hospitalized patients aged 20 to 90 years, diagnosed with CHD and hemodynamically stable, were consecutively recruited from the Department of Cardiology at the Chinese PLA General Hospital [31, 32]. Eligibility for recruitment required a diagnosis of coronary heart disease confirmed through coronary angiography, showing at least one stenosis greater than 50% [33]. We excluded patients who: (1) had conditions such as cardiogenic shock, refractory hypertension, hypertrophic cardiomyopathy, severe valvular heart disease, malignant tumors, severe anemia, myocarditis, and active infections; (2) had a life expectancy of less than one year; (3) were missing key variables. After excluding three heart transplant patients, 175 patients with missing key variables, and 171 lost to follow-up, a total of 3,321 patients were included in the study. The rate of loss to follow-up was 4.9%. The ethics committee of Chinese PLA General Hospital approved this study, and all participants provided written informed consent at the baseline visit.

### TyG index

We extracted fasting triglyceride and blood glucose values, measured at admission, from the electronic medical records. The TyG index was calculated as follows [4]: $TyG\,index=ln \left(\frac{fasting\,triglyceride\,(mg/dL) \times fasting\, glucose\,(mg/dL)}{2} \right)$. We stratified all patients into five groups based on their TyG index quintiles, with each group comprising 664 individuals, except for the first quintile group.

### Covariates

We collected clinical data of the patients, including cardiovascular risk factors, medication usage, and baseline details such as age, gender, vital signs, physical examination, and biochemical parameters. The estimated glomerular filtration rate (eGFR) was calculated using the modified Modification of Diet in Renal Disease (MDRD) equation [34]: $eGFR\,(ml/min/1.73\,m^{2})=175 \times Scr\,(mg/dL) - 1.234 \times Age - 0.179 \times 0.79\,(if\,female).$. The body mass index (BMI) was calculated as follows: $BMI\,(kg/m^{2})=weight\,(kg)\,height^{2}\,(m).$.

### Outcomes and follow-up

The primary outcome of the study was all-cause mortality, while the secondary outcome encompassed major adverse cardiovascular events (MACEs). MACEs were defined as a composite of cardiovascular mortality, myocardial infarction (MI), stroke or transient ischemic attack (TIA), and heart failure (HF) or hospitalization for heart failure (HHF). Telephone follow-ups with all recruited patients occur every two years, with the most recent follow-up deadline in March 2023. Patients without recorded events by this date were considered as right-censored in the analysis.

### Statistical analysis

Continuous variables were expressed as either mean with standard deviation (SD) or median with interquartile range (IQR); categorical variables were presented as count and percentage. Descriptive analyses were conducted using Student’s *t*-test, Mann-Whitney U test, and χ^2^ test, as appropriate.

For the survival analysis, multivariate Cox regression models were utilized to investigate the association between the TyG index and long-term outcomes, including all-cause mortality and MACEs. The TyG index was evaluated as a categorical variable in the multivariable Cox regression model. This model adjusted for age, gender, BMI, smoking, acute coronary syndrome (ACS), previous MI, stroke, hypertension, T2DM, statin, beta blocker, angiotensin-converting enzyme inhibitor (ACE-I)/angiotensin II receptor blocker (ARB), left ventricular ejection fraction (LVEF), eGFR, total cholesterol (TC), and low-density lipoprotein cholesterol (LDL-C). LVEF, TC, and LDL-C underwent natural logarithm transformation prior to inclusion in the models. Nonlinear associations were evaluated using restricted cubic splines to fit smooth curves. Threshold analyses for each model were conducted. The threshold value was determined by exhaustively exploring all possible values, identifying the optimal threshold point based on maximizing the likelihood, and subsequently applying a segmented fit of the Cox regression model.

All statistical analyses were performed using Stata version 17.0 (StataCorp LLC, College Station, TX, USA) and R version 4.1.2 (The R Project for Statistical Computing, Vienna, Austria). A two-tailed *p*-value of < 0.05 was deemed statistically significant.

## Results

### Baseline characteristics

Table 1 presents the baseline characteristics of the 3,321 participants included in the study, stratified by their survival status. The mean age of the study population was 61.7 ± 11.7 years; 72.4% were male; the TyG index ranged from 6.10 to 12.03 with a mean (SD) of 8.9 (0.7). Compared to survivors, deceased patients exhibited higher levels of age, systolic blood pressure (BP), creatinine, FBG, and prevalence of ACS, T2DM, hypertension, previous MI, and stroke. Conversely, deceased patients had lower levels of BMI, diastolic BP, LVEF, eGFR, and fasting TG, along with a decreased prevalence of statin use and current smoking. Baseline characteristics of study participants, stratified by diabetes status, are presented in the additional file (Additional file 1: Table S1).

**Table 1:** Baseline characteristics of study participants stratified by survival status

Baseline characteristics	Total (*n* = 3321)	Survivors^a^ (*n* = 2562)	Non-survivors^a^ (*n* = 759)	p-value
Age, mean (SD), years	61.7 ± 11.7	59.2 ± 10.6	70.4 ± 11.3	< 0.001
Male, n (%)	2404 (72.4%)	1874 (73.1%)	530 (69.8%)	0.073
Current smokers, n (%)	992 (29.9%)	830 (32.4%)	162 (21.3%)	< 0.001
BMI, mean (SD), kg/m²	25.6 ± 3.5	25.9 ± 3.4	24.8 ± 3.8	< 0.001
SBP, mean (SD), mm Hg	135.0 ± 22.3	134.2 ± 22.1	137.9 ± 22.5	< 0.001
DBP, mean (SD), mm Hg	75.7 ± 17.0	76.5 ± 18.1	72.8 ± 12.4	< 0.001
LVEF, median (IQR), %	58.0 (54.0–62.0)	59.0 (55.0–63.0)	55.0 (44.0–59.0)	< 0.001
*Medical history, n (%)*				
Diabetes mellitus	1077 (32.4%)	758 (29.6%)	319 (42.0%)	< 0.001
Hypertension	2175 (65.5%)	1,623 (63.3%)	552 (72.7%)	< 0.001
Previous MI	568 (17.1%)	367 (14.3%)	201 (26.5%)	< 0.001
Stroke	319 (9.6%)	185 (7.2%)	134 (17.7%)	< 0.001
ACS, n (%)	2,479 (74.6%)	1,858 (72.5%)	621 (81.8%)	< 0.001
*Medication, n (%)*				
ACE-I/ARB	1,398 (42.1%)	1,056 (41.2%)	342 (45.1%)	0.060
Beta blocker	2,386 (71.8%)	1,835 (71.6%)	551 (72.6%)	0.600
Statin	3,130 (94.2%)	2,448 (95.6%)	682 (89.9%)	< 0.001
*Laboratory indicators*				
Creatinine, mean (SD), mg/dL	1.0 ± 0.8	0.9 ± 0.5	1.3 ± 1.3	< 0.001
eGFR, mean (SD), mL/min/1.73m^2^	110.5 ± 538.4	116.9 ± 607.9	89.0 ± 144.4	0.210
Glucose, median (IQR), mg/dL	105.8 (90.2–137.5)	104.3 (89.8–132.3)	113.8 (91.8–153.4)	< 0.001
LDL-C, mean (SD), mg/dL	92.7 ± 36.0	93.0 ± 35.9	92.0 ± 36.4	0.530
TC, mean (SD), mg/dL	155.9 ± 41.8	156.1 ± 41.1	155.3 ± 44.1	0.660
TG, mean (SD), mg/dL	144.0 ± 110.5	148.1 ± 115.8	130.3 ± 89.0	< 0.001
TyG index, mean (SD)	8.9 ± 0.7	8.9 ± 0.6	8.8 ± 0.7	0.200

*IQR* inter-quartile range, *SD* standard deviation, *BMI* body mass index, *SBP* systolic blood pressure, *DBP* diastolic blood pressure, *LVEF* left ventricular ejection fraction, *MI* myocardial infarction, *ACS* acute coronary syndrome, *ACE-I* angiotensin-converting enzyme inhibitor, *ARB* angiotensin II receptor blocker, *eGFR* estimated glomerular filtration rate, *LDL-C* low-density lipoprotein cholesterol, *TC* total cholesterol, *TG* triglycerides, *TyG* triglyceride-glucose

^a^Survivors and non-survivors were defined based on whether patients included in the cohort experienced the all-cause mortality during the follow-up period

### Association between TyG index and adverse outcomes

The total follow-up time for 3321 patients with CHD was 29,007.8 person-years (PYs). The median follow-up duration stood at 9.39 (8.70–10.52) years. Throughout the follow-up period, 759 patients encountered all-cause mortality, yielding an incidence rate of 26.17 per 1000 PYs. In addition, 1291 patients experienced MACEs, with an incidence rate of 49.17 per 1000 PYs (Table 2).

**Table 2:** Association between TyG index and adverse outcomes

Variables	Total incidence	Quintiles of TyG index					p for trend
		1st quintile (6.10–8.33)	2nd quintile (8.33–8.67)	3rd quintile (8.67–9.97)	4th quintile (9.97–9.37)	5th quintile (9.37–12.03)	
All-cause mortality							
Events/sample size	759/3321	180/665	157/664	134/664	137/664	151/664	
Incidence per 1,000 PYs (95% CI)	26.17 (24.37–28.09)	31.70 (27.39–36.68)	27.40 (23.44–32.04)	22.77 (19.22–26.97)	23.38 (19.78–27.65)	25.79 (21.99–30.25)	
Crude HR (95% CI)	–	1.40 (1.12–1.75)	1.20 (0.96–1.52)	Ref.	1.03 (0.81–1.31)	1.13 (0.90–1.43)	0.019
Model 1^a^: adjusted HR (95% CI)	–	1.05 (0.84–1.33)	1.03 (0.82–1.29)	Ref.	1.09 (0.85–1.38)	1.47 (1.17–1.86)	0.007
Model 2^b^: adjusted HR (95% CI)	–	1.18 (0.93–1.50)	1.12 (0.89–1.41)	Ref.	1.14 (0.89–1.45)	1.38 (1.07–1.77)	0.270
MACEs							
Events/sample size	1291/3321	281/665	257/664	235/664	235/664	283/664	
Incidence per 1,000 PYs (95% CI)	49.17 (46.56–51.92)	54.76 (48.72–61.55)	49.08 (43.44–55.47)	43.97 (38.70–49.97)	43.75 (38.50–49.72)	54.69 (48.68–61.45)	
Crude HR (95% CI)	–	1.25 (1.05–1.49)	1.12 (0.94–1.34)	Ref.	1.01 (0.83–1.20)	1.25 (1.05–1.49)	0.558
Model 1^a^: adjusted HR (95% CI)	–	1.01 (0.85–1.21)	0.99 (0.83–1.18)	Ref.	1.01 (0.84–1.21)	1.45 (1.22–1.72)	0.001
Model 2^b^: adjusted HR (95% CI)	–	1.10 (0.92–1.31)	1.07 (0.90–1.28)	Ref.	1.02 (0.85–1.23)	1.36 (1.13–1.64)	0.101

*PY* person-year, *HR* hazard ratio, *CI* confidence interval, *MACEs* major adverse cardiovascular events, *TyG* triglyceride-glucose, *ACS* acute coronary syndrome, *ACE-I* angiotensin-converting enzyme inhibitor, *ARB* angiotensin II receptor blocker, *BMI* body mass index, *eGFR* estimated glomerular filtration rate, *LVEF* left ventricular ejection fraction, *TC* total cholesterol, *LDL-C* low-density lipoprotein cholesterol

^a^Model 1 adjusted for age, gender

^b^Model 2 adjusted for age, gender, BMI, smoking, ACS, previous myocardial infarction, stroke, hypertension, diabetes mellitus, statin, beta blocker, ACE-I/ARB, LVEF, eGFR, TC, LDL-C.

The results of the multivariable Cox regression model suggest a potential nonlinear relationship between the TyG index, when included as a categorical variable in the model, and the all-cause mortality and MACEs (Table 2).

### Nonlinear association of TyG index with adverse outcomes

To examine the nonlinear association between the TyG index and adverse outcomes, we employed restricted cubic splines. This was followed by threshold analyses for each model and a segmented fit of the Cox regression model.

**Fig. 1:**
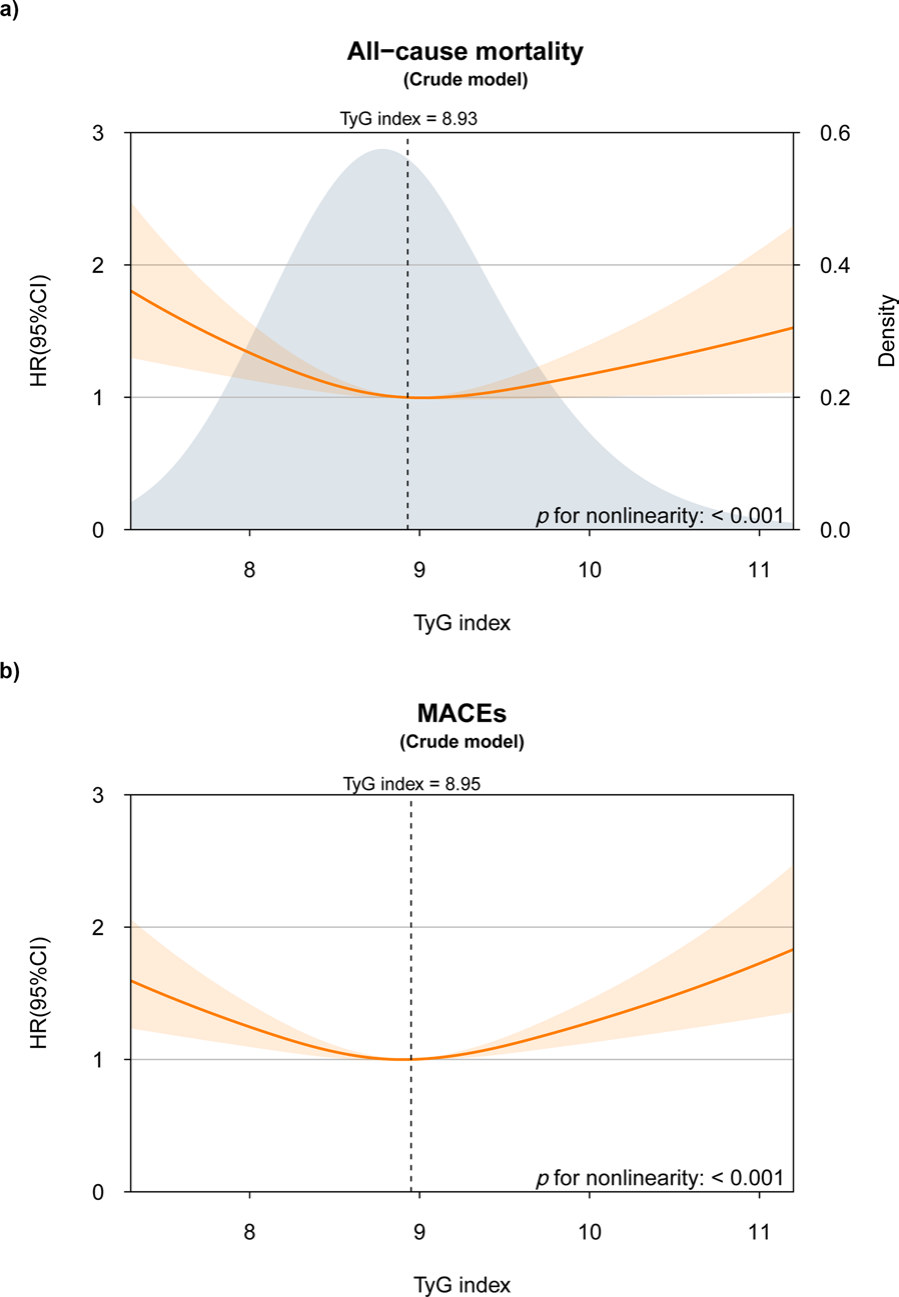
Distribution of TyG index in the study population and the nonlinear association of TyG index with adverse outcomes in the crude model The nonlinear association of TyG index with (**a**) all-cause mortality and (**b**) MACEs

In the crude model, a significant “U”-shaped nonlinear association was observed between the TyG index and both all-cause mortality and MACEs (both *p* for nonlinearity < 0.05; Fig. 1). Threshold analysis identified distinct inflection points for all-cause mortality (TyG index = 8.93) and MACEs (TyG index = 8.95; both *p* for log-likelihood ratio < 0.05). Table 3 presents the segmented fitting results of the Cox regression model.

**Table 3:** Threshold effect analysis of TyG index on all-cause mortality and MACEs

	Crude HR (95% CI)	Adjusted HR^a^ (95% CI)
All-cause mortality		
Total	0.93 (0.83–1.03)	1.14 (1.01–1.29)
Fitting by two-piecewise Cox regression model		
Inflection point	8.93	8.77
TyG index < inflection point (per unit)	0.76 (0.60–0.97)	1.01 (0.73–1.38)
TyG index > inflection point (per unit)	1.37 (1.09–1.72)	1.53 (1.19–1.96)
*p* for Log-likelihood ratio	0.001	0.045
MACEs		
Total	1.02 (0.94–1.11)	1.13 (1.03–1.25)
Fitting by two-piecewise Cox regression model		
Inflection point	8.95	8.62
TyG index < inflection point (per unit)	0.79 (0.65–0.95)	0.71 (0.50–0.99)
TyG index > inflection point (per unit)	1.36 (1.14–1.62)	1.28 (1.10–1.48)
*p* for Log-likelihood ratio	< 0.001	0.009

*HR* hazard ratio, *CI* confidence interval, *MACEs* major adverse cardiovascular events, *TyG* triglyceride-glucose, *ACS* acute coronary syndrome, *ACE-I* angiotensin-converting enzyme inhibitor, *ARB* angiotensin II receptor blocker, *BMI* body mass index, *eGFR* estimated glomerular filtration rate, *LVEF* left ventricular ejection fraction, *TC* total cholesterol, *LDL-C* low-density lipoprotein cholesterol

^a^Adjusted for age, gender, BMI, smoking, ACS, previous myocardial infarction, stroke, hypertension, diabetes mellitus, statin, beta blocker, ACE-I/ARB, LVEF, eGFR, TC, LDL-C

**Fig. 2:**
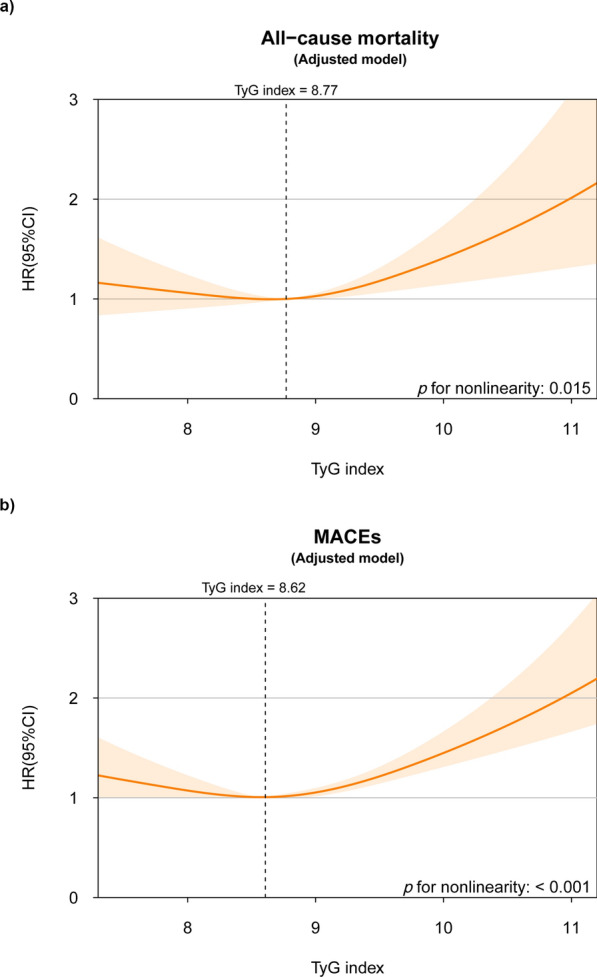
The nonlinear association of TyG index with adverse outcomes in the fully adjusted model. The nonlinear association of TyG index with (**a**) all-cause mortality and (**b**) MACEs. Spline curves were adjusted for age, gender, BMI, smoking, ACS, previous myocardial infarction, stroke, hypertension, diabetes mellitus, statin, beta blocker, ACE-I/ARB, LVEF, eGFR, TC, LDL-C

In the fully adjusted model, the TyG index maintained its significant “U”-shaped nonlinear association with MACEs (TyG index < 8.62: per unit increase, HR 0.71, 95% CI 0.50–0.99; TyG index > 8.62: per unit increase, HR 1.28, 95% CI 1.10–1.48; *p* for log-likelihood ratio = 0.009; Fig. 2; Table 3). Additionally, the TyG index continued to exhibit a significant nonlinear association with all-cause mortality (*p* for nonlinearity = 0.015). However, threshold analysis revealed significance only when the TyG index exceeded 8.77, indicating a higher risk of all-cause mortality (per unit increase, HR 1.53, 95% CI 1.19–1.96). Below this threshold, the TyG index was not significantly associated with all-cause mortality (per unit increase, HR 1.01, 95% CI 0.73–1.38) (Table 3).

**Fig. 3:**
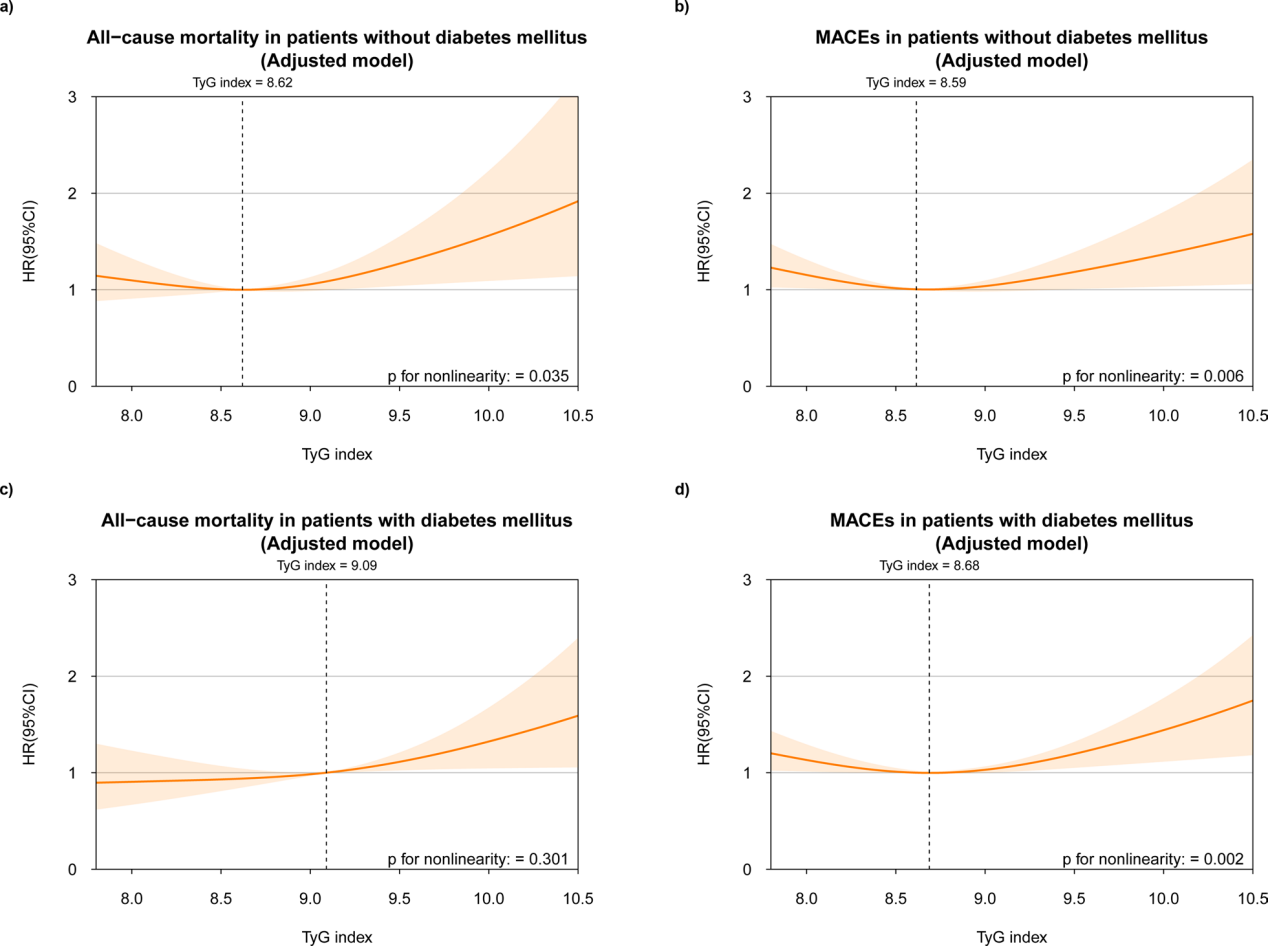
The nonlinear association of the TyG index with adverse outcomes in the fully adjusted model for both diabetic and non-diabetic patients. The nonlinear association of TyG index with (**a**) all-cause mortality and (**b**) MACEs in non-diabetic patients. The nonlinear association of TyG index with (**c**) all-cause mortality and (**d**) MACEs in diabetic patients. Spline curves were adjusted for age, gender, BMI, smoking, ACS, previous myocardial infarction, stroke, hypertension, diabetes mellitus, statin, beta blocker, ACE-I/ARB, LVEF, eGFR, TC, LDL-C. When the nonlinear association is significant, the reference point is the inflection point; otherwise, it is the median of the TyG index

We also investigated the nonlinear relationship between the TyG index and adverse outcomes among diabetic and non-diabetic patient subgroups in the multivariate model. In both subgroups, a “U”-shaped association was noted between the TyG index and MACEs (nonlinear *p* < 0.05; Fig. 3b, d). In non-diabetic patients, a nonlinear association was observed between the TyG index and all-cause mortality (nonlinear *p* = 0.035; Fig. 3a), with an increased risk of mortality at a TyG index above the inflection point. Conversely, in diabetic patients, there was a linear relationship between the increase in the TyG index and the risk of all-cause mortality (nonlinear *p* = 0.301; Fig. 3c).

Additionally, we have presented the relationships between triglycerides and glucose with all-cause mortality and MACEs (Additional file 1: Fig. S1), as well as the relationship between TyG index and the components of MACEs (Additional file 1: Fig. S2) in Figures in the appendix.

## Discussion

In this study, we have for the first time identified a significant nonlinear association between the baseline TyG index and increased risk of all-cause mortality and MACEs over an extended period in hospitalized patients with CHD. The results from the threshold analysis revealed a distinct inflection point in the association between TyG index and all-cause mortality and MACEs, where TyG index demonstrated a U-shaped association with MACEs. Beyond the inflection point, an elevated TyG index correlated with an increased risk of all-cause mortality.

Previous clinical studies have linked the TyG index with CVD in the general population without a history of CVD [7–9]. This correlation has been observed in various groups, including middle-aged and elderly individuals, adolescents, individuals with and without T2DM, populations at high risk for CVD, and postmenopausal women [10–15]. Among CVD patients, an elevated TyG index has been associated with an increased risk of major adverse cardiovascular and cerebrovascular events in individuals with stable CHD combined with T2DM [16]. Numerous studies have independently confirmed the association of the TyG index with MACEs in patients with ACS, irrespective of T2DM status [17–23]. In our study, we also noted that an elevated TyG index beyond 8.77 and 8.62 was significantly associated with increased risks of both all-cause mortality and MACEs. Further examination of the nonlinear association between the TyG index and adverse outcomes in both diabetic and non-diabetic patients showed a transition to a linear association in diabetic patients, where an increase in the TyG index correlated with an increased risk of all-cause mortality. The observed change in the association may arise from the smaller number of patients with CHD combined with T2DM included in this study.

The precise biological mechanism connecting the TyG index to adverse cardiovascular events in patients with CHD remains elusive. As a reliable surrogate for IR, the primary mechanism behind the negative outcomes associated with a heightened TyG index could be related to IR. Patients with IR typically exhibit a heightened risk of metabolic disorders, such as hyperglycemia, dyslipidemia, and hypertension, all closely linked to negative CVD outcomes [35–37]. Additionally, IR can cause increased platelet activity and elevated adhesion-induced expression of thromboxane A2-dependent tissue factors in platelets, leading to thrombosis and inflammation [38]. This may partly explain the occurrence of ischemic events observed in CHD patients, including MI and stroke. Furthermore, prolonged IR can lead to enhanced activity of the sympathetic nervous system and renal sodium retention, resulting in higher blood pressure and increased cardiac afterload [39, 40]. Chronic hyperglycemia and dyslipidemia caused by IR can induce oxidative stress, exacerbate inflammatory responses, impair endothelial function, and promote the proliferation of smooth muscle cells and collagen deposition. These factors might contribute to cardiac fibrosis and eventually heart failure [22, 41].

Previous research has indicated that extremely low levels of TG or FBG are linked to adverse health outcomes and could precipitate disease [42]. The results of this study also demonstrate a similar trend (Additional file, Figure S1). Hypoglycemia has been associated with an increased risk of cardiovascular events or cerebrovascular stroke [43]. Similarly, low TG levels have been recognized as a predictive factor for cardiac mortality in patients with heart failure [44]. Prior studies have demonstrated a significant relationship between a reduction in the TyG index within a specific range and a heightened risk of adverse events [28–30]. In our study, a significant association was found between a reduction in the TyG index and an increased long-term risk of MACEs in CHD patients when the TyG index fell below 8.62. A similar trend was noted in the association between the TyG index and all-cause mortality, although the statistical significance of this trend might be limited by the sample size and residual confounders.

Previous research indicates that an elevated TyG index is associated with an increased risk of major adverse cardiovascular and cerebrovascular events in individuals with stable CHD combined with T2DM [16]. Numerous studies have independently confirmed the association of the TyG index with MACEs in diabetic patients with CHD [18, 24, 45–47]. As for non-diabetic patients with CHD, Zhao et al. showed that the TyG index may serve as a useful predictive marker for MACEs in the non-diabetic population with ACS patients [25]. Our study results reveal that the TyG index is not only associated with MACEs in CHD patients but also exhibit a “U”-shaped nonlinear relationship. However, some researchers present different perspectives. A cohort study involving 5,489 non-diabetic patients who underwent percutaneous coronary intervention (PCI) indicated that the TyG index is not an effective predictive factor for adverse cardiovascular prognosis in non-diabetic patients undergoing PCI [48]. A similar conclusion was also published in another cohort study, suggesting that the TyG index should not be used as a predictor of MACEs and all-cause mortality among non-diabetic patients with MI at a 1-year follow-up [49]. Currently, there are no other research reports the nonlinear relationship between TyG index and MACEs. However, other researchers have identified a “U”-shaped nonlinear association between the TyG index and all-cause mortality and cardiovascular mortality through threshold analysis [28–30]. This finding suggests that the potential protective or harmful effects of different TyG index levels on either side of the inflection points might counterbalance within the integrated model. This counterbalance could explain why some researchers have inferred a lack of significant correlation between the TyG index and adverse outcomes [26, 27, 48, 49].

This study also explored the correlation between the TyG index and each component of MACEs in CHD patients (Additional file, Figure S2). We observed that cardiovascular mortality in patients with CHD showed an elevated risk when the TyG index exceeded the inflection point, mirroring the trend observed in all-cause mortality. This differs from the “U”-shaped nonlinear correlation of the TyG index with cardiovascular mortality and all-cause mortality found by Zhang et al. in CVD patients [28]. Additionally, we identified a “U”-shaped nonlinear correlation between the TyG index and long-term HF/HHF in patients with CHD.

Previous studies have not explored the predictive value of the TyG index for long-term adverse outcomes in hospitalized patients with CHD. The unique contribution of this study is its identification of a nonlinear relationship between the TyG index and long-term adverse outcomes in a substantial cohort of hospitalized patients with CHD, particularly noting the “U”-shaped relationship between the TyG index and subsequent MACEs. Nevertheless, this study is not without limitations. Firstly, as a single-center study involving adults in China, its findings might not extend to other demographic groups. Secondly, despite efforts to control confounding variables, eliminating all residual confounding factors affecting prognosis, such as nutritional and socioeconomic status, is unattainable due to the lack of relevant variables. Thirdly, the study did not account for the dynamic changes in the TyG index of patients. Future research should focus on the dynamic changes in patients’ TyG index and their association with adverse outcomes, as well as interventions targeting the TyG index to improve clinical outcomes.

Our study presented evidence of a nonlinear association between the TyG index and long-term adverse outcomes in both diabetic and non-diabetic populations with CHD. Upon further recognition of the clinical utility of the TyG index in the future, it could be automatically calculated by a computer based on the patient’s measured fasting glucose and triglycerides, similar to eGFR (calculated by the computer based on creatinine), providing clinicians with greater convenience in guiding clinical decisions.

## Conclusion

This study revealed a nonlinear relationship between the TyG index and both all-cause mortality and MACEs among hospitalized patients with CHD. Assessing the TyG index, particularly focusing on individuals with extremely low or high TyG index values, could improve risk assessment for adverse outcomes in this group. Future studies are warranted to investigate interventions that target the TyG index to enhance clinical outcomes.

### Supplementary Information

**Additional file 1: Table S1.** Baseline characteristics of study participants stratified by diabetes status. **Figure S1**. The association of triglycerides and glucose with long-term adverse outcomes in hospitalized patients with CHD. **Figure S2**. The association between TyG index and each component of long-term MACEs in hospitalized patients with CHD.

## Data Availability

No datasets were generated or analysed during the current study.

## Abbreviations

ACE-I: Angiotensin-converting enzyme inhibitor
ACS: Acute coronary syndrome
ARB: Angiotensin II receptor blocker
BMI: Body Mass Index
BP: Blood pressure
CHD: Coronary heart disease
CI: Confidence interval
CVD: Cardiovascular disease
eGFR: Estimated glomerular filtration rate
FBG: Fasting blood glucose
HF: Heart failure
HHF: Hospitalization for heart failure
HR: Hazard ratio
IQR: Interquartile range
IR: Insulin resistance
LDL-C: Low-density lipoprotein cholesterol
LVEF: Left ventricular ejection fraction
MACEs: Major adverse cardiovascular events
MDRD: Modification of diet in renal disease
MI: Myocardial infarction
PCI: Percutaneous coronary intervention
PYs: Person-years
SD: Standard deviation
T2DM: Type 2 diabetes mellitus
TC: Total cholesterol
TG: Triglycerides
TIA: Transient ischemic attack
TyG: Triglyceride-glucose
