# Supplementary material for: The relationship between triglyceride-glucose index and prospective key clinical outcomes in patients hospitalised for coronary artery disease
Source: Cardiovasc Diabetol. 2024 Jan 22;23:40. doi: 10.1186/s12933-024-02132-2 (PMC10804527; doi:10.1186/s12933-024-02132-2)

**The Relationship between Triglyceride-Glucose Index and Prospective Key Clinical Outcomes in Patients Hospitalised for Coronary Artery Disease**

**Contents:**

1. Table S1. Baseline characteristics of study participants stratified by diabetes status.
2. Figure S1. The association of triglycerides and glucose with long-term adverse outcomes in hospitalized patients with CHD.
3. Figure S2. The association between TyG index and each component of long-term MACEs in hospitalized patients with CHD.

**Table S1. Baseline characteristics of study participants** **stratified** **by diabetes status**

| **Baseline characteristics** | **Total**  **(n = 3,321)** | **Diabetic patients**  **(n = 1,077)** | **Non-diabetic patients**  **(n = 2,244)** | ***p*-value** |
| --- | --- | --- | --- | --- |
| Age, mean (SD), years | 61.7±11.7 | 62.4±11.4 | 61.4±11.9 | 0.016 |
| Male, n (%) | 2,404 (72.4%) | 749 (69.5%) | 1,655 (73.8%) | 0.011 |
| Current smokers, n (%) | 992 (29.9%) | 279 (25.9%) | 713 (31.8%) | <0.001 |
| BMI, mean (SD), kg/m² | 25.6±3.5 | 26.0±3.4 | 25.5±3.6 | <0.001 |
| SBP, mean (SD), mm Hg | 135.0±22.3 | 137.2±20.2 | 134.0±23.1 | <0.001 |
| DBP, mean (SD), mm Hg | 75.7±17.0 | 75.3±11.6 | 75.9±19.1 | 0.310 |
| LVEF, median (IQR), % | 58.0 (54.0–62.0) | 58.0 (53.0–61.0) | 58.0 (54.0–62.0) | 0.001 |
| *Medical history, n (%)* |  |  |  |  |
| Hypertension | 2,175 (65.5%) | 787 (73.1%) | 1,388 (61.9%) | <0.001 |
| Previous MI | 568 (17.1%) | 198 (18.4%) | 370 (16.5%) | 0.170 |
| Stroke | 319 (9.6%) | 115 (10.7%) | 204 (9.1%) | 0.150 |
| ACS, n (%) | 2,479 (74.6%) | 804 (74.7%) | 1,675 (74.6%) | 0.999 |
| *Medication, n (%)* |  |  |  |  |
| ACE-I/ARB | 1,398 (42.1%) | 521 (48.4%) | 877 (39.1%) | <0.001 |
| Beta blocker | 2,386 (71.8%) | 800 (74.3%) | 1,586 (70.7%) | 0.031 |
| Statin | 3,130 (94.2%) | 1,011 (93.9%) | 2,119 (94.4%) | 0.520 |
| *Laboratory indicators* |  |  |  |  |
| Creatinine, mean (SD), mg/dL | 1.0±0.8 | 1.0±0.9 | 1.0±0.7 | 0.001 |
| eGFR, mean (SD), mL/min/1.73m^2^ | 110.5±538.4 | 102.1±127.1 | 114.5±649.1 | 0.530 |
| Glucose, median (IQR), mg/dL | 105.8 (90.2–137.5) | 133.4 (106.9–188.3) | 98.1 (87.7–118.8) | <0.001 |
| LDL-C, mean (SD), mg/dL | 92.7±36.0 | 89.9±34.6 | 94.1±36.6 | 0.002 |
| TC, mean (SD), mg/dL | 155.9±41.8 | 152.8±41.3 | 157.4±42.0 | 0.003 |
| TG, mean (SD), mg/dL | 144.0±110.5 | 153.5±116.4 | 139.5±107.3 | <0.001 |
| TyG index, mean (SD) | 8.9±0.7 | 9.1±0.7 | 8.7±0.6 | <0.001 |

IQR, inter-quartile range; SD, standard deviation; BMI, body mass index; SBP, systolic blood pressure; DBP, diastolic blood pressure; LVEF, left ventricular ejection fraction; MI, myocardial infarction; ACS, acute coronary syndrome; ACE-I, angiotensin-converting enzyme inhibitor; ARB, angiotensin II receptor blocker; eGFR, estimated glomerular filtration rate; LDL-C, low-density lipoprotein cholesterol; TC, total cholesterol; TG, triglycerides; TyG, triglyceride-glucose.

**Figure S1. The association of triglycerides and glucose with long-term adverse outcomes in hospitalized patients with CHD**

The association of triglycerides with **a)** all-cause mortality and **b)** MACEs, and glucose with **c)** all-cause mortality and **d)** MACEs in hospitalized patients with CHD.

The variables in the multivariable model include triglycerides, glucose, age, gender, BMI, smoking, ACS, previous myocardial infarction, stroke, hypertension, diabetes mellitus, statin, beta-blocker, ACE-I/ARB, LVEF, eGFR, TC, LDL-C. When the nonlinear association is significant, the reference point is the inflection point; otherwise, it is the median of triglycerides or glucose.


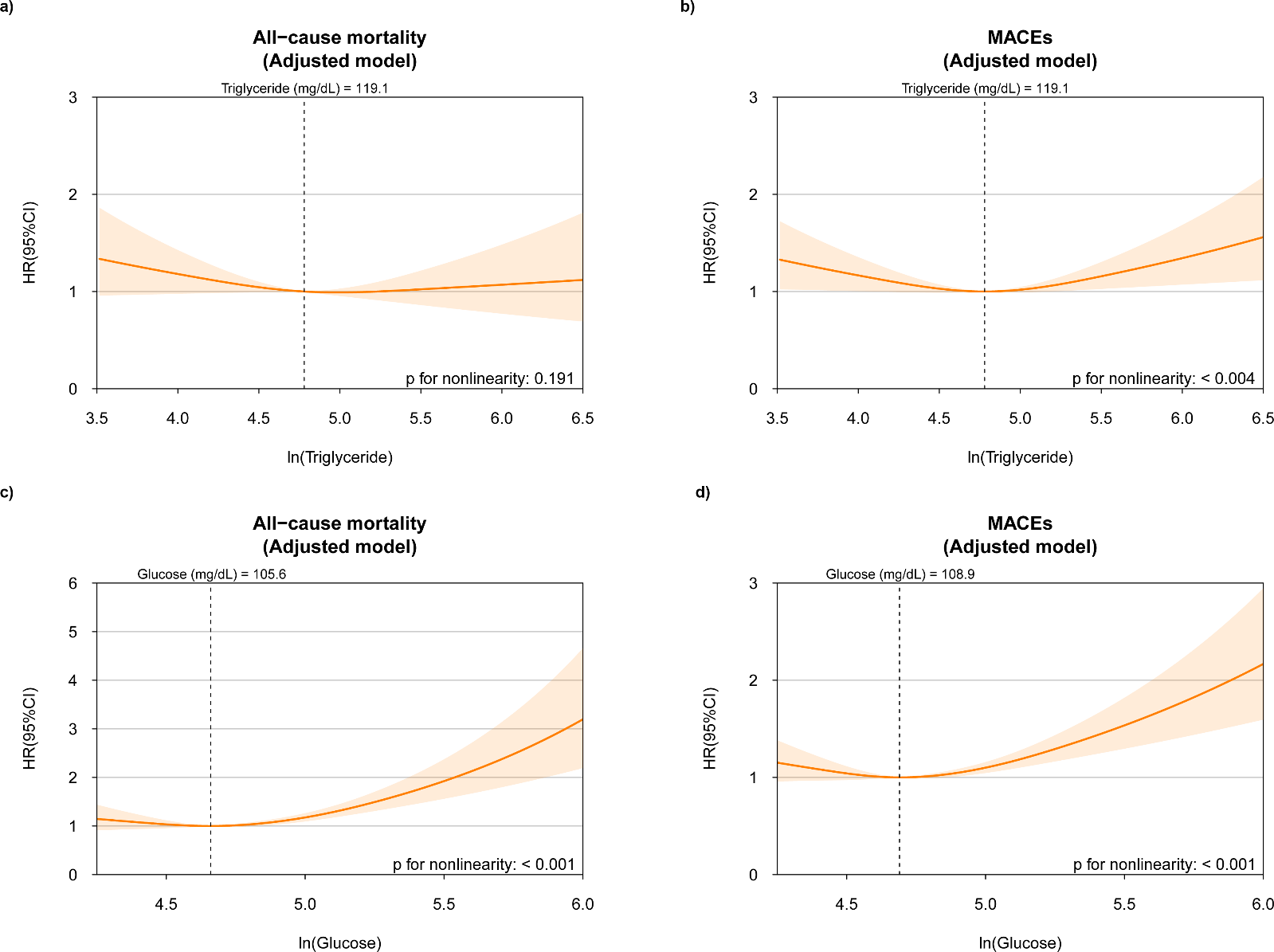


**Figure S2. The association between TyG index and each component of long-term MACEs in hospitalized patients with CHD**

The nonlinear association of the TyG index with **a)** cardiovascular mortality, **b)** myocardial infarction, **c)** stroke/ transient ischemic attack, and **d)** heart failure/ hospitalization for heart failure in hospitalized patients with CHD.

Spline curves were adjusted for age, gender, BMI, smoking, ACS, previous myocardial infarction, stroke, hypertension, diabetes mellitus, statin, beta blocker, ACE-I/ARB, LVEF, eGFR, TC, LDL-C. When the nonlinear association is significant, the reference point is the inflection point; otherwise, it is the median of the TyG index.
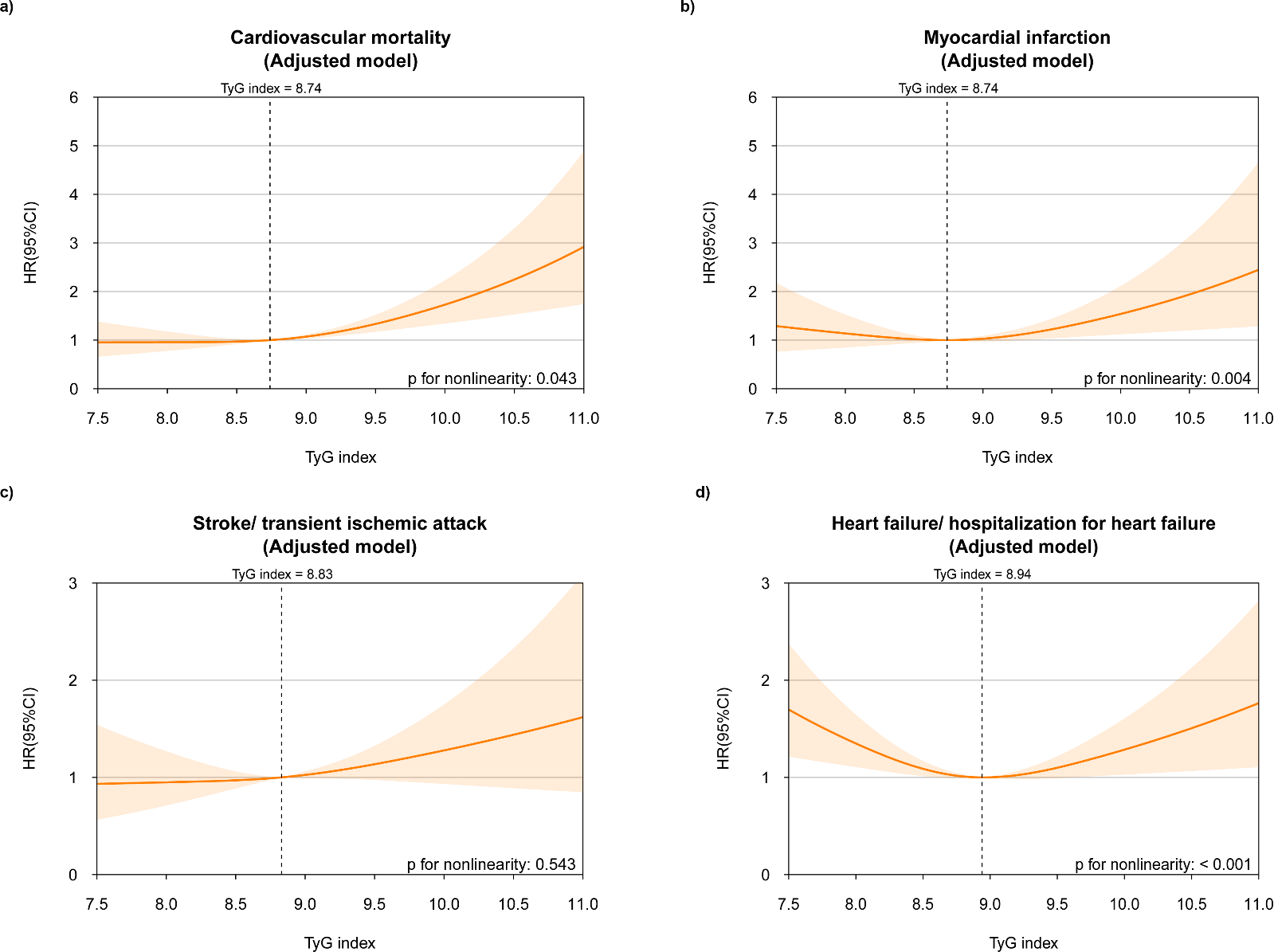

Supplement: Supplementary file 1 — Additional file 1: Table S1. Baseline characteristics of study participants stratified by diabetes status. Figure S1. The association of triglycerides and glucose with long-term adverse outcomes in hospitalized patients with CHD. Figure S2. The association between TyG index and each component of long-term MACEs in hospitalized patients with CHD. [file 12933_2024_2132_MOESM1_ESM.docx]
